# Author Correction: Liquid metal droplets bouncing higher on thicker water layer

**DOI:** 10.1038/s41467-024-46487-2

**Published:** 2024-03-07

**Authors:** Yuhang Dai, Minfei Li, Bingqiang Ji, Xiong Wang, Siyan Yang, Peng Yu, Steven Wang, Chonglei Hao, Zuankai Wang

**Affiliations:** 1 Department of Mechanical Engineering, City University of Hong Kong, Hong Kong, 999077 China; 2 Department of Mechanical and Aerospace Engineering, Southern University of Science and Technology, Shenzhen, 518055 China; 3 School of Mechanical Engineering and Automation, Harbin Institute of Technology, Shenzhen, 518055 China; 4 Department of Mechanical Engineering, Hong Kong Polytechnic University, Hong Kong, 999077 China

**Keywords:** Fluid dynamics, Wetting

**Correction to**: *Nature Communications* 10.1038/s41467-023-39348-x, published online 14 June 2023

The original version of this Article contained an error in Fig. 1. In the original version of Fig. 1a, the snapshots start from liquid metal droplets sticking to substrate which is not consistent with what the authors described in the article. The order of snapshots 4 and 5 in Fig. 1b was reversed. The correct version of Fig. 1 is:
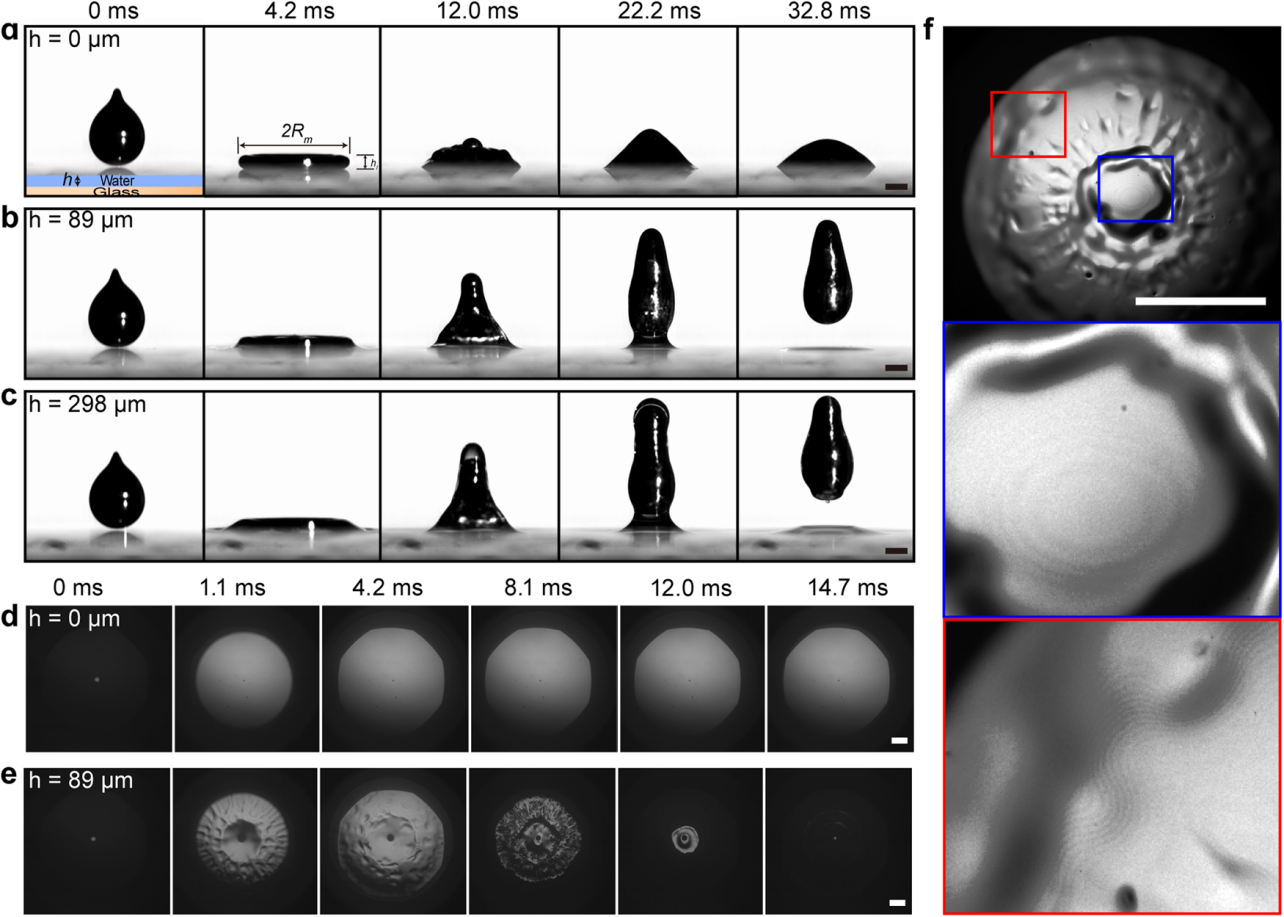


which replaces the previous incorrect version:
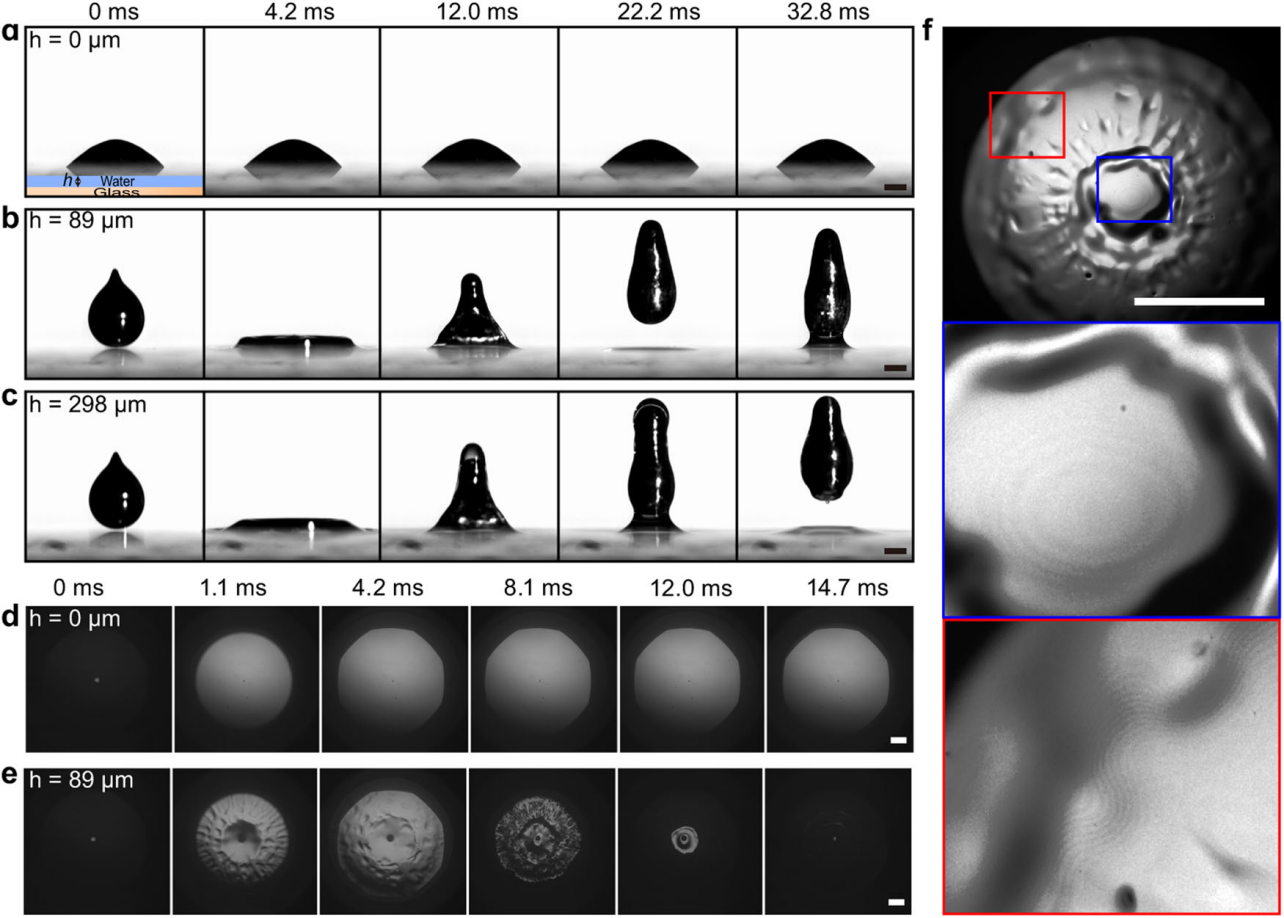


In the original version of this Article, Eq. (2) contained two typographical errors, which was incorrectly written as:

$$e={\left\{\frac{1}{2}-{C}_{1}\cdot \frac{1}{2}\frac{3}{2W{{{\rm{e}}}}}\cdot \frac{{\gamma }_{{{{\rm{wa}}}}}}{{\gamma }_{{{{\rm{ma}}}}}}\cdot \frac{1}{H}-{C}_{2}\cdot \frac{3\pi }{10}\cdot W{e}^{10/9}O{h}_{{{{\rm{f}}}}}^{1/9}{\left(\frac{{\gamma }_{{{{\rm{mw}}}}}}{{\gamma }_{{{{\rm{ma}}}}}}\right)}^{-11/18}\right\}}^{0.5}$$

The correct equation should be written as:

$$e={\left\{\frac{1}{2}-{C}_{1}\cdot \frac{3}{2W{{{\rm{e}}}}}\cdot \frac{{\gamma }_{{{{\rm{wa}}}}}}{{\gamma }_{{{{\rm{ma}}}}}}\cdot \frac{1}{H}-{C}_{2}\cdot \frac{3}{10\pi }\cdot W{e}^{10/9}O{h}_{{{{\rm{f}}}}}^{1/9}{\left(\frac{{\gamma }_{{{{\rm{mw}}}}}}{{\gamma }_{{{{\rm{ma}}}}}}\right)}^{-11/18}\right\}}^{0.5}$$

These errors have been corrected in both the PDF and HTML versions of the Article.

