# Supplementary material for: Liquid metal droplets bouncing higher on thicker water layer
Source: Nat Commun. 2023 Jun 14;14:3532. doi: 10.1038/s41467-023-39348-x (PMC10267135; doi:10.1038/s41467-023-39348-x)
Supplement: Supplementary file 1 — Supplemntary Information [file 41467_2023_39348_MOESM1_ESM.docx]

**Supplementary Information: Liquid metal droplets bouncing higher on thicker water layer**

Yuhang Dai^1,3#^, Minfei Li^1#^, Bingqiang Ji^1#^, Xiong Wang^1^, Siyan Yang^1^, Peng Yu^3^, Steven Wang^1*^, Chonglei Hao^4*^, Zuankai Wang^2,1*^

^1^Department of Mechanical Engineering, City University of Hong Kong, Hong Kong 999077, China

^2^ Department of Mechanical Engineering, Hong Kong Polytechnic University, Hong Kong 999077, China

^3^Department of Mechanical and Aerospace Engineering, Southern University of Science and Technology, Shenzhen 518055, China

^4^School of Mechanical Engineering and Automation, Harbin Institute of Technology, Shenzhen 518055, China.

*steven.wang@cityu.edu.hk (S. W.); *haoc@hit.edu.cn (C.H.); *zk.wang@polyu.edu.hk (Z.W.)

# Y. D., B. J., and M. L. contributed equally to this work.

**Supplementary Figures**


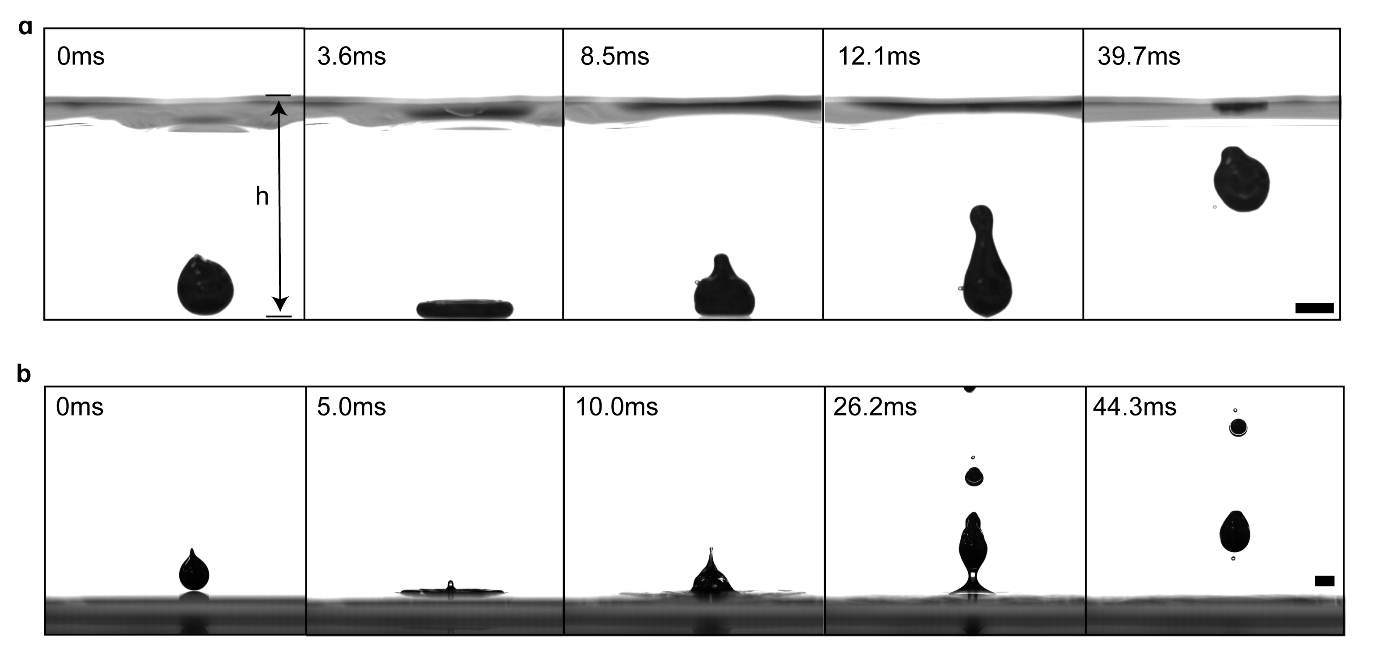


**Supplementary Figure S1** | Experimental snapshots of bare LM droplet bouncing off a water layer-covered glass slide. (a) We = 10.7, *H* = 6.7 (*h* = 10 mm). (b) We = 26.8, *H* = 0.25 (*h* = 0.38 mm). The droplet radius *R* = 1.5 mm. The scale bar is 2 mm.


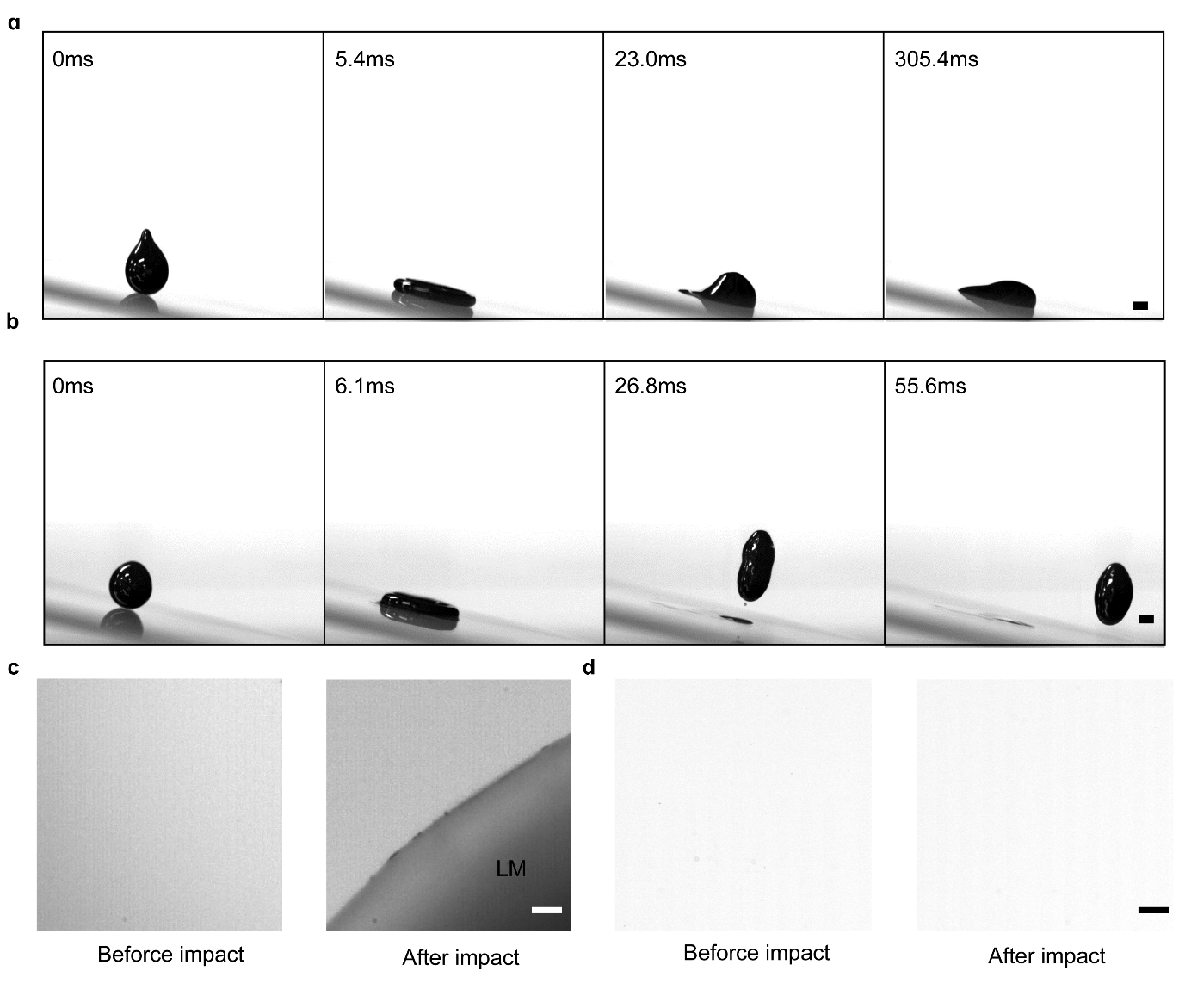


**Supplementary Figure S2** | (a) Selected snapshots of a bare LM droplet impacting on an inclined glass surface and (b) a core-shell LM droplet impacting on an inclined surface with the thickness of the bottom shell *h*_s_ = 55 μm. The inclination angle of the glass slides is 13.5°, and the scale bar is 1 mm in (a) and (b). (c) and (d) the microscope images of the impact area at the glass slide surface before (left) and after (right) impact in (a) and (b), respectively, with a magnification of 10X. The images clearly show that the LM droplet sticks to the glass slide upon impact without the presence of water (c), while there are no LM residuals on the glass slide, i.e., no contact between the LM droplet and the glass slide during impact with the presence of a thin water shell layer (d). The scale bar is 0.1 mm in (c) and (d).


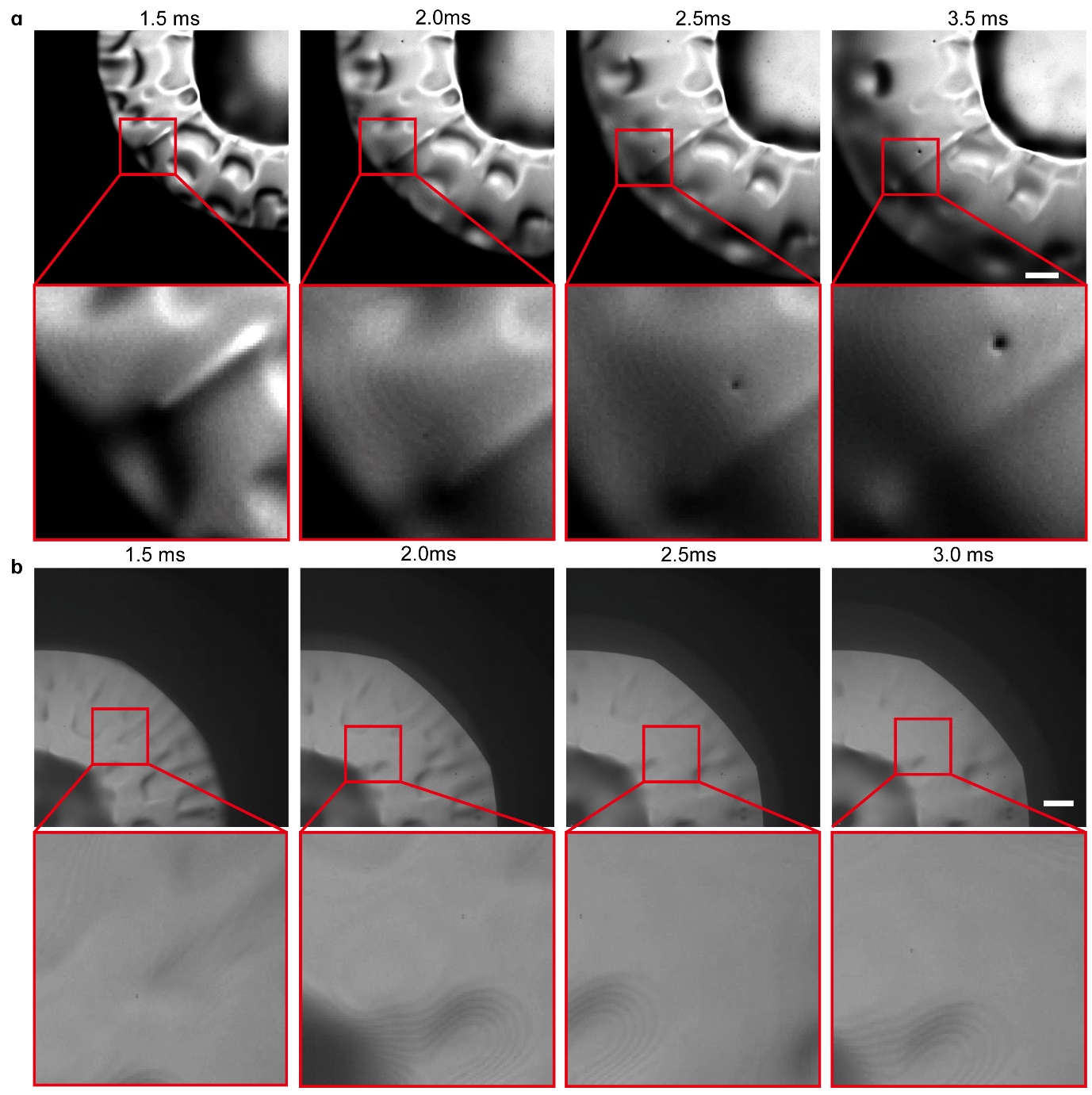


**Supplementary Figure S3** | Interference fringes imaged by the RICM for bare LM droplet impacting on water layer-covered glass slide. (a)*We* = 10.72, *H* = 0.2. (b) *We* = 10.72, *H* = 0.06. The scale bar is 0.2 mm.


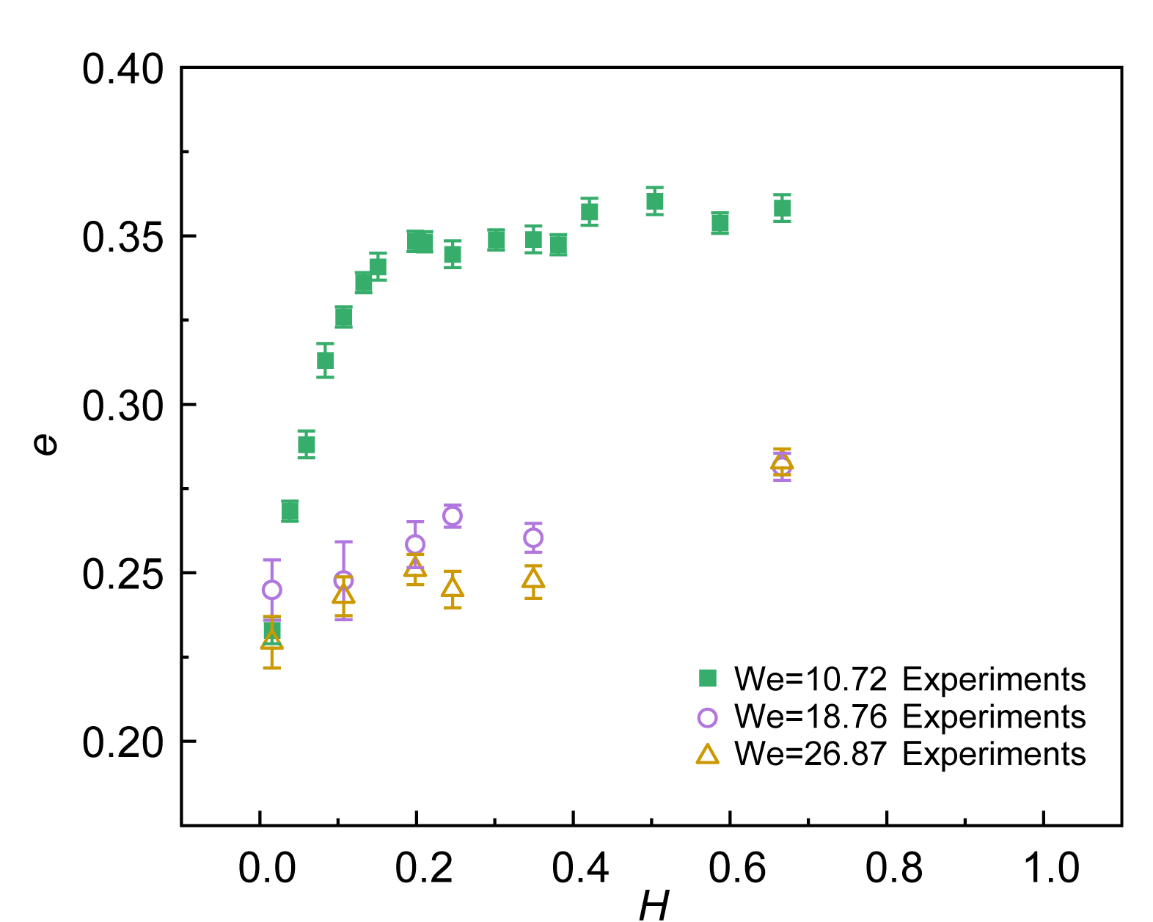


**Supplementary Figure S4** | Restitution coefficient *e* as a function of dimensionless water layer thickness *H* for bare LM droplet impacting on water layer-covered glass slides with different *We*. The scatters at large *We* (18.8 and 26.8) may be caused by the energy loss of the ejected small jet droplets due to the droplet breakup at the retraction stage.


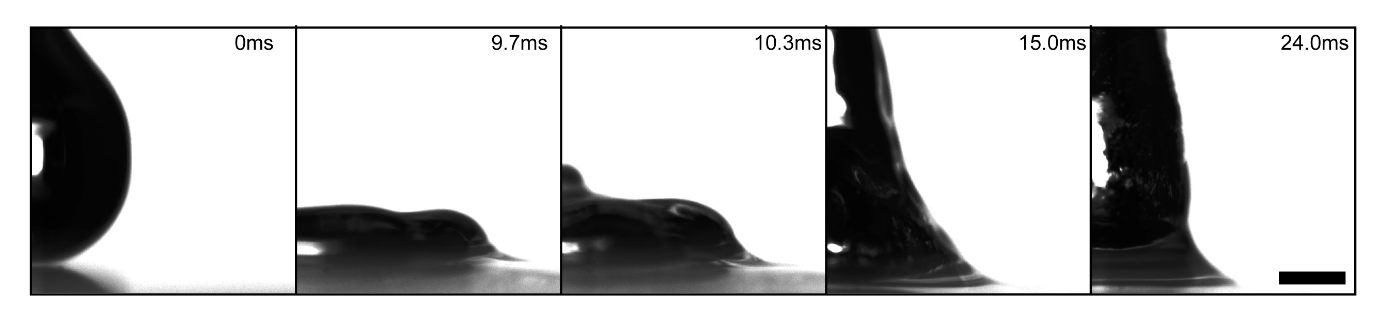


**Supplementary Figure S5 |** Experimental images showing that the water meniscus forms at the edge of the LM droplet due to the fast spreading of the water layer on the LM surface and persists during the retraction stage. *We* = 10.7, *H* = 0.1. The scale bar is 1mm.


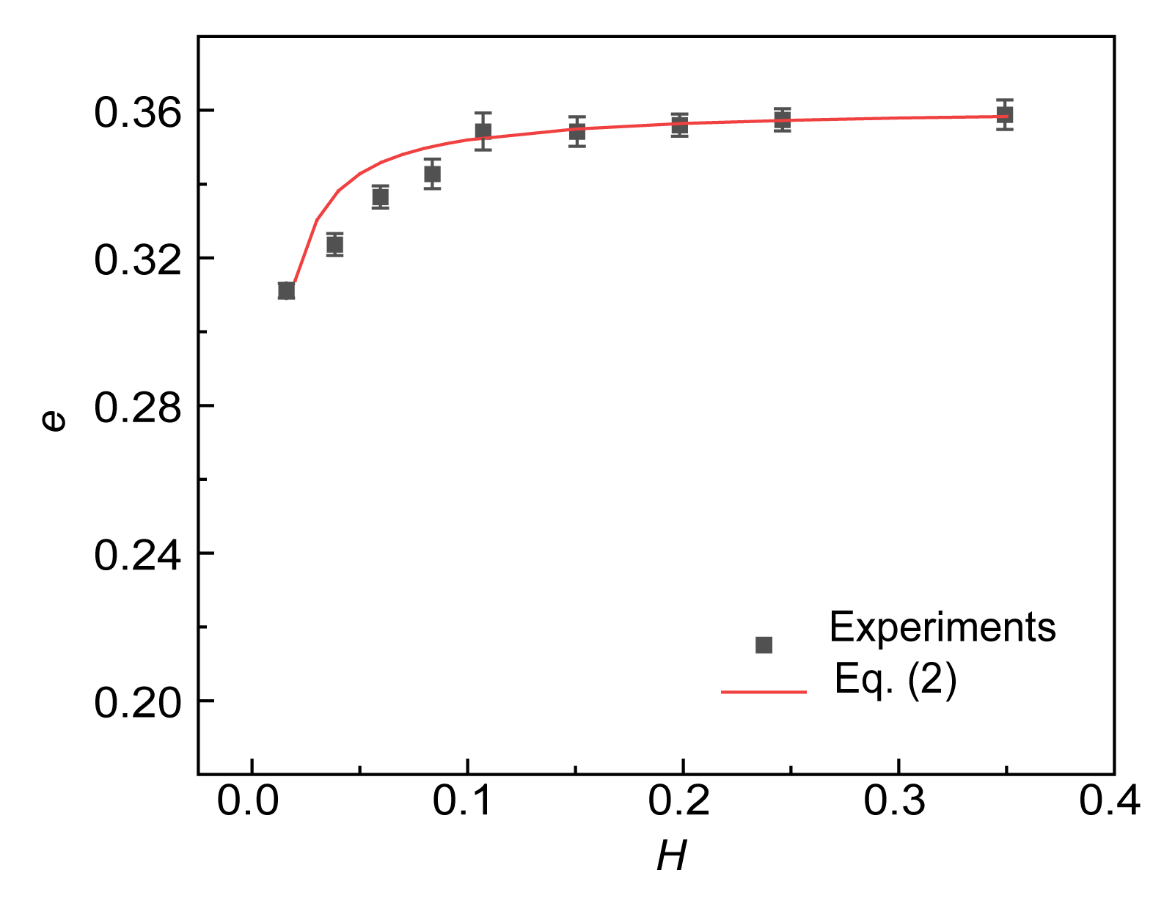


**Supplementary Figure S6** | Restitution coefficient *e* as a function of dimensionless liquid layer thickness *H* for bare LM droplets impacting on a glass slide covered by a surfactant aqueous liquid layer of 3.5 mM/L sodium dodecyl benzene sulfonate. Here *We* = 10.7, *γ*_wa_ = 0.031 mN/m and *γ*_mw_ = 0.484 mN/m. The theoretical predictions using Eq. (2) agree well with the experimental results.


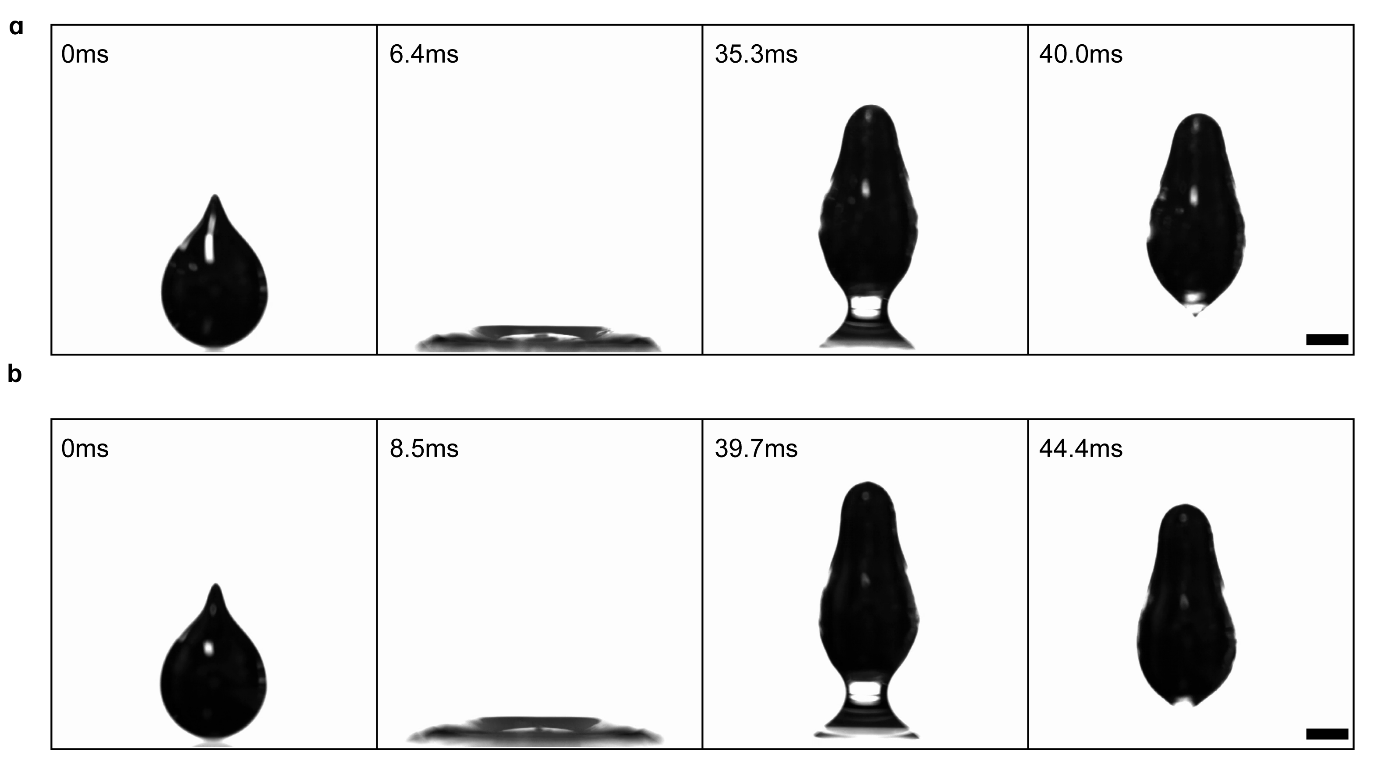


**Supplementary Figure S7 |** Selected snapshots of high-speed camera images showing the bouncing of LM droplet impinging on a glass slide covered by a 10 wt% glycerin aqueous solution layer. (a) *We* = 10.7, *H* = 0.1 (b) *We* = 10.7, *H* = 0.15. The scale bar is 1 mm.


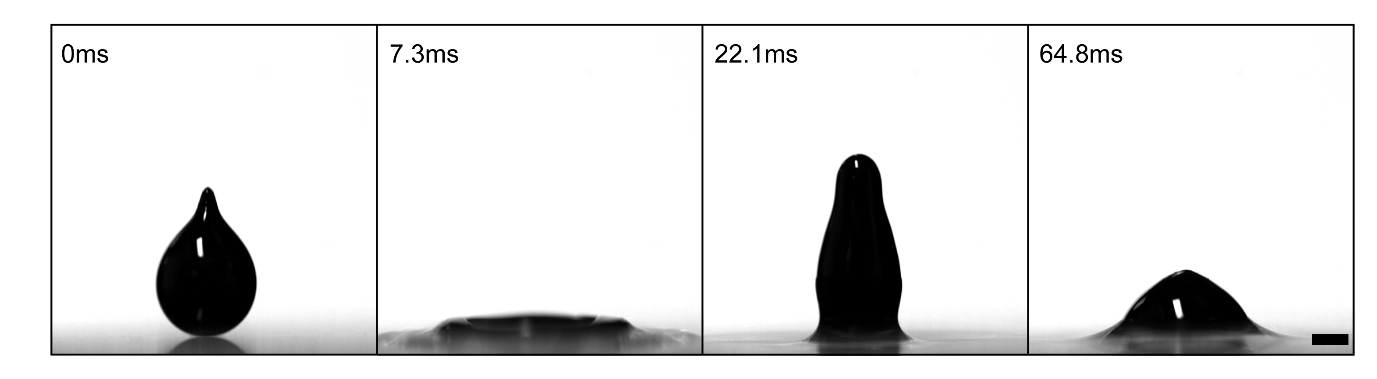


**Supplementary Figure S8 |** Selected snapshots of high-speed camera images showing that the LM droplet cannot bounce off after impinging on a glass slide covered by a highly viscous liquid layer (60 wt% glycerin aqueous solution with a viscosity of 0.01 Pa s). We = 10.7, *H* = 0.1. The scale bar is 1 mm.

**Supplementary movies**

**Supplementary Movie 1** | The impact dynamics of LM droplet (volume ~14 μL) on a glass substrate with different water thickness *h* of 0 μm (left), 89 μm (middle), and 298 μm (right) at *We* = 10.7. The frame rate set is 6000 frames per second using a high-speed camera (Photron, FASTCAM SA4).

**Supplementary Movie 2** | The bottom view videos of LM droplet (volume ~14 μL) impacting on a glass substrate with different water thickness *h* of 0 μm (left) and 89 μm (right) at *We* = 10.7. The image is captured by the inverted microscope with a laser imaging system coupled with a high-speed camera.

**Supplementary Movie 3** | The interference fringes imaged by RICM during a bare LM droplet impacting on a water layer-covered glass slide at *We* = 10.7, *H* = 0.06. The scale bar is 0.2mm.
