# Supplementary material for: Liquid metal droplets bouncing higher on thicker water layer
Source: Nat Commun. 2023 Jun 14;14:3532. doi: 10.1038/s41467-023-39348-x (PMC10267135; doi:10.1038/s41467-023-39348-x)
Supplement: Supplementary file 3 — Description of Additional Supplementary Files [file 41467_2023_39348_MOESM3_ESM.pdf]

Description of Additional Supplementary Files

File name: Supplementary movies1

Description: The impact dynamics of LM droplet (volume ~14 μL) on a glass substrate with different water thickness *h* of 0 μm (left), 89 μm (middle), and 298 μm (right) at *We* = 10.7. The frame rate set is 6000 frames per second using a high-speed camera (Photron, FASTCAM SA4). The scale bar is 1mm.

File name: Supplementary movies2

Description: The bottom view videos of LM droplet (volume ~14 μL) impacting on a glass substrate with different water thickness *h* of 0 μm (left) and 89 μm (right) at *We* = 10.7. The image is captured by the inverted microscope with a laser imaging system coupled with a high-speed camera. The scale bar is 1mm.

File name: Supplementary movies3

Description: The interference fringes imaged by RICM during a bare LM droplet impacting on a water layer-covered glass slide at *We* = 10.7, *H* = 0.06. The scale bar is 1mm.
